# The Vocabulary of Infants with an Elevated Likelihood and Diagnosis of Autism Spectrum Disorder: A Systematic Review and Meta-Analysis of Infant Language Studies Using the CDI and MSEL

**DOI:** 10.3390/ijerph19031469

**Published:** 2022-01-27

**Authors:** Zsofia Belteki, Raquel Lumbreras, Kloe Fico, Ewa Haman, Caroline Junge

**Affiliations:** 1 Department of Experimental Psychology, Helmholtz Institute, Utrecht University, 3584 CS Utrecht, The Netherlands; c.m.m.junge@uu.nl; 2 Faculty of Medicine, Utrecht University, 3584 CS Utrecht, The Netherlands; raquelldlf@gmail.com; 3 Donders Institute for Brain, Cognition and Behavior, Radboud University, 6525 XZ Nijmegen, The Netherlands; kloe.fico@donders.ru.nl; 4 Faculty of Psychology, University of Warsaw, 00-927 Warsaw, Poland; ewa.haman@psych.uw.edu.pl

**Keywords:** infancy, autism, vocabulary, CDI, MSEL

## Abstract

Diagnoses of autism spectrum disorder (ASD) are typically accompanied by atypical language development, which can be noticeable even before diagnosis. The siblings of children diagnosed with ASD are at elevated likelihood for ASD diagnosis and have been shown to have higher prevalence rates than the general population. In this paper, we systematically reviewed studies looking at the vocabulary size and development of infants with autism. One inclusion criterion was that infants were grouped either pre-diagnostically as elevated or typical likelihood or post-diagnostically as ASD or without ASD. This review focused on studies that tested infants up to 24 months of age and that assessed vocabulary either via the parent-completed MacArthur–Bates Communicative Developmental Inventory (CDI) or the clinician-administered Mullen Scales of Early Learning (MSEL). Our systematic search yielded 76 studies. A meta-analysis was performed on these studies that compared the vocabulary scores of EL and TL infants pre-diagnostically and the scores of ASD and non-ASD infants post-diagnostically. Both pre- and post-diagnostically, it was found that the EL and ASD infants had smaller vocabularies than their TL and non-ASD peers, respectively. The effect sizes across studies were heterogenous, prompting additional moderator analyses of age and sub-group analyses of the language measure used (CDI or MSEL) as potential moderators of the effect size. Age was found to be a moderator both in the pre- and post-diagnostical groups, however, language measure was not a moderator in either diagnostic group. Interpretations and future research directions are discussed based on these findings.

## 1. Introduction

Autism spectrum disorder (ASD) is a developmental condition accompanied by impairments in social communication and interaction, as well as restrictive and repetitive behaviours or interests [1]. This includes differences in how the vocabularies of ASD infants develop compared to their non-ASD peers [2,3].

Some aspects of social communication and interaction are shown to be affected in ASD infants even prior to diagnosis. Infants who have an older sibling with ASD have an increased probability of receiving a diagnosis by approximately 20%, relative to the general population [4]. The behavioural and cognitive profiles of these infants with an ‘elevated likelihood’ (EL) of ASD diagnosis can shed light on how the developmental condition manifests prior to diagnosis. In existing literature, while some studies have classified infants solely on the basis of their genetic background (i.e., prospectively), other studies classify infants after diagnosis (i.e., retrospectively). In prospective studies, two groups can be identified: elevated likelihood (EL) and typical likelihood (TL). In retrospective studies, infants can be classified as ASD, non-ASD, or as developing with other conditions such as language delay.

Vocabulary can be defined as the words that the infant understands (or is receptive to) and the words they are able to speak (or express) [5]. Vocabulary development has been observed to be affected both in prospective and retrospective studies. In prospective studies, EL infants have been shown to have smaller expressive and/or receptive vocabulary compared to their TL peers [6,7,8,9,10,11,12,13,14,15].

However, there are mixed findings regarding the extent to which expressive and receptive vocabulary develop of EL and TL infants differ and (if so) in which age groups these differences in development are observable. For example, when comparing EL and TL receptive vocabularies, Iverson et al. [6] reported significant differences in vocabulary scores of EL and TL infants by 11 months, whereas Nyström et al. [16] did not find significant differences between the EL and TL groups for expressive or receptive vocabulary at 10 months [16]. Similarly, other studies also found that EL infants did not differ in their receptive and expressive vocabulary sizes from TL peers [17,18].

There are a number of methodological differences that may factor into the mixed findings that we observe in the literature. One reason for mixed findings may be variations across studies in the age of testing. Across the first two years of life, there are changes in how large the differences are between the infant groups in their expressive and receptive vocabulary sizes. For example, longitudinal assessments of infants have found larger differences between older compared to younger EL and TL infant groups for both expressive and receptive vocabulary [6]. Additionally, for expressive vocabulary, it has been observed that although the expressive vocabulary size of EL and TL infants is similar at 6 months of age, by 12 months, EL infants are observed to produce fewer words than their TL peers [19]. In light of these findings, it is important to assess how age impacts group differences between EL and TL and ASD and non-ASD infants so that we can more clearly map the expressive and receptive vocabulary development of the developmental groups.

Another factor that may impact group differences is the language measure that is used in a study. There are multiple standardised assessment tools available for evaluating the expressive and receptive vocabulary of infants in the first years of life. Two standardised assessments that are frequently used to assess the vocabulary of ASD and non-ASD infants are the MacArthur–Bates Communicative Development Inventory (CDI) [5] and the Mullen Scales of Early Learning; both are available in multiple languages [20,21]. Both the CDI and MSEL assess expressive and receptive vocabulary but in different ways. The CDI assesses infants’ vocabulary through a word checklist that is completed by parents. Depending on the age of administration, either the ‘Words and Gestures’ CDI (around 300 words) or the ‘Words and Sentences’ (around 700 words) of the CDI is used. The MSEL assesses vocabulary by directly testing infants. It has an expressive scale made up of 28 items and a receptive scale made up of 33 items, which includes checks such as whether the infant ‘coordinates listening and looking’. The test is carried out by clinicians who are trained on how to assess expressive and receptive vocabulary. There are therefore multiple differences between the CDI and MSEL in how and by who they are carried out, which can impact the assessment of language that they make.

The influence of language measures on vocabulary outcomes has been examined less frequently than the effect of age but may be worth considering for a few reasons [22,23]. Firstly, contextual factors, such as how tired or motivated a child is to participate can have an impact on the vocabulary scores they receive. Contextual factors may have more impact on the scoring in the MSEL than the CDI, because the MSEL is completed in one sitting of 5–15 min, while the CDI can be completed outside of a fixed time frame and in multiple contexts (e.g., at home or school). Although previous studies have observed high correlations between the CDI and MSEL, these observations were made when assessing children [22]. When assessing infants, correlations between the CDI and MSEL may be lower because contextual factors are shown to have a larger impact in younger children [22]. Therefore, the scores that infants receive on questionnaires and assessments may vary more across contexts than the scores that children receive. This increased variability in the scores received across contexts may also impact how the scores of an infant compare when they are assessed on the one assessment versus another, i.e., the CDI versus the MSEL.

An additional reason as to why language measurement may have an impact on the vocabulary outcomes that are observed is differences in how reliably clinicians versus parents can assess the infant. The CDI is administered by parents who do not received standardised training on how to complete the questionnaire. In contrast, the MSEL is administered by clinicians who are trained on how to assess infant expressive and receptive vocabulary. Parents may, due to lack of training, be less able to reliably determine when their child understands and/or produces a word [23,24,25]. On the other hand, parents may also be more acquainted with the words that their infants understand and speak than a clinician who interacts with the child for only a brief period of time. Subsequently, we may expect differences between parental and clinician scorings of vocabulary due to differences in familiarity with the infant and the training received or not received. To discern the magnitude of differences more accurately between EL and TL and ASD and non-ASD infants’ vocabularies, we need to better understand the extent to which differences in vocabulary scores derive from the assessment tool used.

This paper aimed to systematically review and meta-analyse the existing literature, looking at the expressive and receptive vocabulary sizes of elevated likelihood (EL) and ASD infants between the age groups of 0 and 24 months. Both studies comparing infants prospectively (i.e., comparing EL and TL infants) and retrospectively (i.e., comparing ASD versus non-ASD diagnosed) were included in this review. Focusing on the earliest stages of vocabulary acquisition (i.e., from 0–24 months of age) allows us to compare the results of studies that classify infants prospectively versus retrospectively, because after 24 months of age, infants belonging to the ‘elevated’ and ‘typical’ likelihood groups often receive diagnoses as ASD or non-ASD. In comparing the results of retrospective and prospective studies, we aimed to further our understanding on how infants classified as ‘elevated likelihood’ (that is, infants with a genetic background of ASD but no diagnosis) differ in their receptive and expressive profiles from infants who go on to receive a diagnosis of ASD (infants with a genetic background and also a diagnosis). Pre-diagnostically, the EL group contains a larger variation of developmental profiles than the post-diagnostic group of infants who are diagnosed with ASD. Roughly 80% of EL infants receive no diagnosis or are diagnosed with a different developmental disorder from ASD. The relatively small proportion of infants in the EL group that receive an ASD diagnosis warrants investigation of whether the group differences observed pre-diagnostically between EL and TL infants are comparable to the group differences observed post-diagnostically between ASD and non-ASD infants.

In this paper, we focused on studies that compare the vocabularies of the infant groups using the CDI and the MSEL as their language measures. Although there are other measures which can be used to assess infants’ vocabulary, such as the Bayles Scales of Infant and Toddler Development [26], we chose to focus on the CDI and MSEL because they are regularly used as language assessments for ASD and EL populations, both in individual studies and in larger infant cohorts [27,28,29,30]. Although both measures collect data on expressive and receptive vocabulary, their methodologies differ—whereas the CDI is parent completed and a questionnaire, the MSEL is clinician completed and involves infants completing a set of tasks. By focusing on these measures in the meta-analysis, we aimed to assess whether their differing methodologies impacts the results that are obtained in the studies. This impact of methodology has previously been assessed for the CDI and MSEL, but not in 0–24-month-old infants [22]. Gaining a better understanding of the impact of methodology on language outcomes could be important information for large infant cohorts that test infants on both the CDI and MSEL.

In this paper, we hypothesised that EL and ASD infants will generally have smaller expressive and receptive vocabulary sizes compared to their typical likelihood (TL) and non-ASD peers. We predict this effect to become more pronounced with age, with group differences between EL versus TL and also ASD versus non-ASD infants increasing with age [31]. We also hypothesised that the method of language measure will moderate the group differences that are observed between EL versus. TL infants and also between ASD versus non-ASD infants. We hypothesise this because it can be more challenging to reliably assess the vocabularies of younger compared to older children. Subsequently, the method of language measure is more likely to impact the assessment that is made of the infant.

## 2. Materials and Methods

### 2.1. Systematic Review

#### 2.1.1. Search Strategy

The systematic search was carried out in December 2020 on the following search engines: PubMed, Psych Info, and Google Scholar. Search queries were adapted for each of these databases. The databases were chosen so that studies could be located on the from a medical database (PubMed) and then also from a more psychological database (Psych Info). Google Scholar was additionally used as a database to ensure access to studies that may have fallen outside of the formerly mentioned disciplines. The exact search terms are specified in Table 1. It should be noted that Google Scholar returned records classified by relevance, and therefore we limited the review of Google Scholar results to 200 records.

#### 2.1.2. Screening Process

All records were collected in Zotero and assessed for inclusion by one of the authors (R.L.). Before the inclusion assessment, duplicated records were detected and removed using Zotero. The inclusion of records was decided on the basis of the predefined criteria specified in Table 2. All titles and abstracts were first screened using Rayyan [32], a tool specifically designed for this purpose. The reports that did not meet inclusion criteria were discarded, and the remaining records were selected for full-text assessment. In case of doubts about the inclusion of a paper, a decision was taken in discussion with two other authors (Z.B. and C.J.).

#### 2.1.3. Literature Search

**Figure 1:**
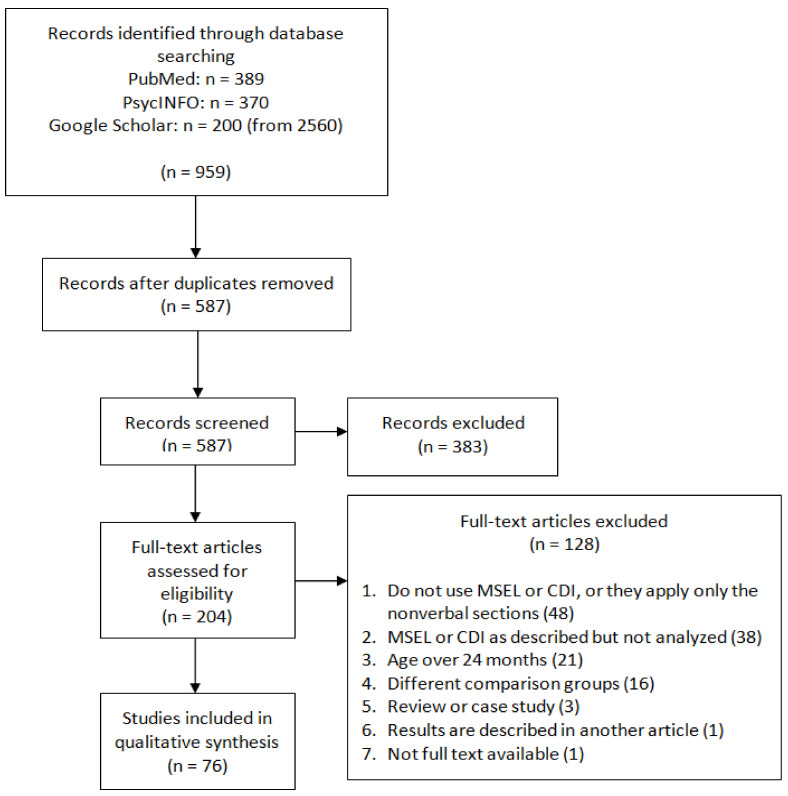
PRISMA flow diagram of the systematic review [33].

The search resulted in 389 records from PubMed, 370 from PsycINFO, and approximately 2560 from Google Scholar. Only the first 200 results were selected from Google Scholar because this search engine organises records by relevance. Therefore, a total of 959 records were retrieved from the three databases. From these records, 372 duplicated records were removed, and the remaining 587 were screened for their title and abstract. During the screening, 383 records were excluded for not meeting the inclusion criteria, and 204 were selected for full-text assessment. Full-text reading resulted in 76 final articles that met the inclusion criteria and were included in this review. The assessment and exclusion of records are illustrated in Figure 1.

### 2.2. Meta-Analysis

#### 2.2.1. Inclusion and Exclusion Criteria

In the follow-up meta-analyses, we focused solely on the studies from the systematic review that compared verbal scores of pre-diagnostic EL and TL infants and/or post-diagnostic ASD and non-ASD groups. This excluded the studies that compared non-verbal scores (e.g., gestures), leaving a total of 65 studies from the systematic review. These studies can be found in Table A1, Table A2, Table A3 and Table A4 in Appendix A.

#### 2.2.2. Data Extraction

The articles from the systematic review were compiled in a template taken from Metalab [34,35]. From each paper, the following categories of information were extracted: paper description, such as publication year, and experiment description, such as the age groups that were tested and then information to calculate effect size. We also sought out information regarding other measures that were collected on the infants (such as language background) but were not able to locate this in a number of studies. The relevant data from the papers were compiled independently by three coders (K.F., R.L., Z.B.). Each of the coders extracted approximately one-third of the total reports. At the start of the coding, inter-coder reliability was confirmed by comparing the entries of the three coders on one study.

#### 2.2.3. Meta-Analytic Procedure

Meta-analyses were run in excel and R-studio version 1.4. 1103 using resources from the MetaLab website and the Meta-essentials workbooks [34,36]. The meta-analyses were run on the pre-diagnostic datasets, i.e., where infants were classified as elevated likelihood (EL) or typical likelihood (TL), as well as on the post-diagnostic datasets, i.e., where infants were classified on the basis of a diagnosis of ASD or non-ASD. Expressive and receptive vocabulary were separately assessed in both pre- and post-diagnostic datasets.

The effect sizes were derived through the difference in the scores obtained by the developmental groups, either pre-diagnostically (i.e., EL compared to TL) or post-diagnostically (i.e., ASD compared to non-ASD). Effect sizes were calculated as the magnitude of difference in the scores of the infant groups. Pre-diagnostically, this was calculated as TL scores minus EL scores. A positive value meant that the EL group scored lower on expressive or receptive vocabulary assessments than their TL peers. Post-diagnostically, this was calculated as non-ASD score minus ASD infant score. A positive value meant that the ASD group scored lower on expressive or receptive vocabulary assessments than their non-ASD peers.

Studies with effect sizes above 3 were removed to make the meta-analyses more conservative. Then, in both in the pre- and post-diagnostic samples, the weighted mean effect size, which is the average effect size of all the studies, was calculated.

Additionally, the heterogeneity was calculated, which is the magnitude of variance across studies in effect sizes. Data that are heterogenous contains sub-domains with different ‘true’ effect sizes. Heterogeneity is calculated by first calculating Cochran’s Q, which is the weighted sum of differences between the observed differences and the average effect size, and then comparing this Q-statistic with the variation that would be observed if all studies were from the same population. Heterogeneity is interpreted through the I^2^ value, which is a percentage that explains what proportion of the variance is explained by real differences in effect size. If the studies are found to be heterogenous and the I^2^ percentage is high, then it is worthwhile to explore the heterogeneity in moderator analyses and/or sub-group analyses [37].

Moderator analyses were planned (if there was a large heterogeneity) to observe if there was a relationship between the age of the infants and the effect size that was found in the studies. This was done through a regression analysis.

Sub-group analyses were planned (if there was a large heterogeneity) to assess whether there are two sub-groups in a domain that have a different weighted ‘true’ effect size. Studies were compared on the basis of the language measure (CDI or MSEL) they used. Differences in the true effect size of CDI versus MSEL studies were compared through a between-factor ANOVA.

Studies were also checked for publication bias, which is concerned with a selection bias that might occur after studies have been conducted, specifically a bias in the studies that were published versus not published [37]. The underlying hypothesis is that studies that have statistically significant results are more likely to be published than studies with non-significant results. Publication bias is assessed using funnel plots and Egger regression. The analyses detect the presence of a publication bias and also adjust the weighted mean effect size accordingly (however this weighted mean effect size is only interpretable if the studies’ effect sizes are homogenous).

## 3. Results

Below, we list our meta-analyses, first for pre-diagnostic groups. For both pre-diagnostic and post-diagnostic groups, we report both expressive and receptive outcomes, respectively. Interpretations of the results were guided by meta-analytic resources [37,38].

### 3.1. Pre-Diagnostic Results

#### 3.1.1. Expressive Vocabulary

The weighted mean effect over the whole dataset was significantly above zero, with Cohen’s *d* = 0.36 [0.27, 0.45], *SE* = 0.05. The weighted mean effect, which is positive and significantly above zero, indicates that EL infants scored lower on expressive vocabulary than their TL peers.

***Publication bias.*** Following the removal of all effect sizes above 3, there was no salient evidence of bias, with the data spreading symmetrically around the mean. The Egger test was non-significant (*p* = 0.67) for publication bias.

***Heterogeneity.*** Heterogeneity was significant, *Q*(36) = 74.32, *p* < 0.001; total heterogeneity *I*^2^ = 50.22%. Considering this, we turned to testing whether effect size differed depending on age (moderator analyses) and assessment used (sub-group analyses).

***Moderator analyses: Is the main effect size influenced by the age of the infants?***

**Figure 2:**
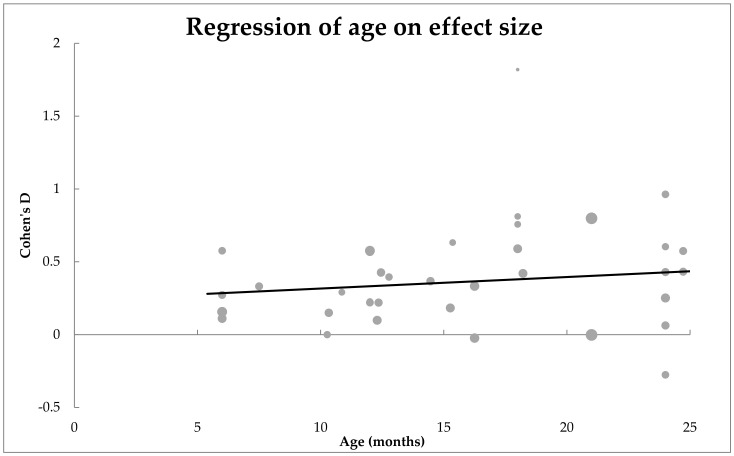
Scatterplot of how age moderated the mean difference between EL and TL infants’ expressive vocabulary. No significant effect of moderator was observed.

Age was not found to be a significant moderator of effect size, *n* = 36, *QM*(1) = 0.70, *p* = 0.40. These results are shown in Figure 2 as a scatter plot. Estimates for Cohen’s *d* were mostly positive for all age groups, but it was not statistically significant from zero, *β* = 0.17, SE = 0.01. This lack of correlation suggests that the mean difference between the EL and TL infant groups did not increase with age.

***Sub-group analyses: Is the main effect size dependent on the assessment (CDI or MSEL) used?*** The effect sizes of the sub-groups of the language measure (CDI and MSEL) did not significantly differ (*n* = 36, *QM*(1) = 0.29, *p* = 0.59). The CDI group showed small effects (Cohen’s *d* = 0.31; [0.17, 0.45]) that were homogeneous (*I*^2^ = 0.00%). In contrast, the MSEL group also showed small effect sizes (Cohen’s *d* = 0.37 [0.25, 0.48]), but they were heterogenous (*I*^2^ = 61.55%).

#### 3.1.2. Receptive Vocabulary

The weighted mean effect over the whole dataset was significantly above zero, with Cohen’s *d* = 0.42 [0.29, 0.55], *SE* = 0.06. The weighted mean effect, which was positive and significantly above zero, indicated that EL infants scored lower on receptive vocabulary than their TL peers.

***Publication bias.*** Following the removal of all effect sizes above 3, there was no salient evidence of publication bias, with the data spreading symmetrically around the mean. The Egger test was non-significant (*p* = 0.72) for publication bias.

***Heterogeneity.*** Heterogeneity was significant, *Q*(32) = 88.49, *p* < 0.001; total heterogeneity *I*^2^ = 63.84%. Considering this, we turned to the focus of the paper: to test the influence of age (moderator analyses) and assessment used (sub-group analyses).

**Figure 3:**
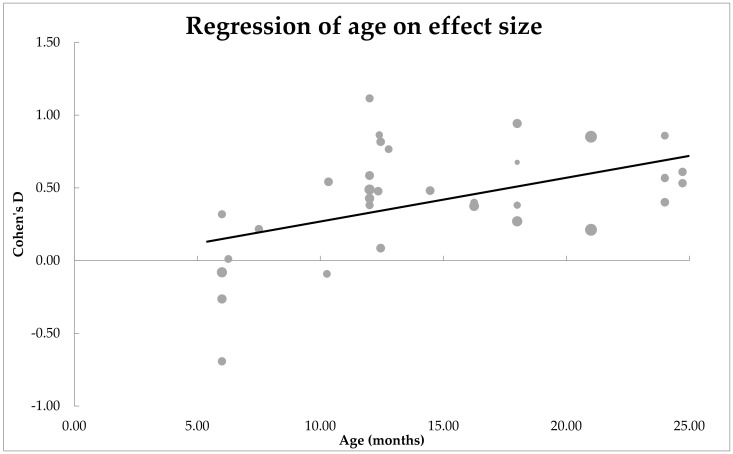
Scatterplot of how age moderated the mean difference between EL and TL infants’ receptive vocabulary. A significant effect of moderator was observed.

***Moderator analyses: Is the main effect size influenced by the age of the infants?*** Age was found to be a significant moderator of effect size, *n* = 32, *QM*(1) = 8.55, *p* = 0.003. These results are shown in Figure 3 as a scatter plot. Estimates for Cohen’s *d* were mostly positive for all age groups and were statistically significant from zero, *β* = 0.50, *SE* = 0.01. The positive correlation suggests that the mean difference between the EL and TL infants increases with age.

***Sub-group analyses: Is the main effect size dependent on the assessment (CDI or MSEL) used?*** The effect sizes of the sub-groups of language measure (CDI and MSEL) did not significantly differ, *n* = 32, *QM*(1) = 1.19, *p* = 0.28. The CDI group was somewhat heterogeneous (*I*^2^ = 4.19%), with no true main effect size (Cohen’s *d* = 0.53, [0.32, 0.74]). The MSEL group was also heterogeneous (*I*^2^ = 69.24%), (Cohen’s *d* = 0.38, [0.24, 0.53]).

#### 3.1.3. Summary of Results: Pre-Diagnostic Groups

Overall, there were moderate effect size observed for the studies. The direction of this effect size was that the EL infants had smaller receptive and expressive vocabularies than their TL peers.

Age was not found to be a moderator for expressive vocabulary. In contrast, for receptive vocabulary, age was a moderator of the effect size observed—as age increased, the mean difference between the EL and TL infants increased, with the gap in the receptive vocabulary size of the EL and TL infant groups becoming increasingly larger.

For neither expressive nor receptive vocabulary, significant differences on effect sizes depended on the assessment tool (CDI or MSEL) used. The language measure, therefore, found similar differences between the EL and TL infants across studies. However, for expressive vocabulary, it was found that the CDI studies were homogenous, i.e., had a true effect size. This true effect size was 0.31, which is a moderate effect. There was more heterogeneity in effect sizes from the studies using the MSEL.. Further investigation is needed looking at the factors feeding into the MSEL groups’ heterogeneity.

### 3.2. Post-Diagnostic Results

#### 3.2.1. Expressive Vocabulary

The weighted mean effect over the whole dataset was significantly above zero, with Cohen’s *d* = 0.89 [0.65, 1.13], *SE* = 0.12. The weighted mean effect, which is positive and significantly above zero, indicates that infants with ASD scored lower on expressive vocabulary than their TD peers.

***Publication bias.*** Following the removal of all effect sizes above 3, there was still a salient evidence of publication bias. The Egger test was significant (*p* = 0.035) for publication bias.

***Heterogeneity.*** Heterogeneity was significant, *Q*(35) = 115.40, *p* < 0.001; total heterogeneity *I*^2^ = 77.48%. Considering this, we turned to the focus of the paper: to test the influence of age and assessment used on the expressive vocabularies of infants diagnosed with ASD compared to TD infants.

**Figure 4:**
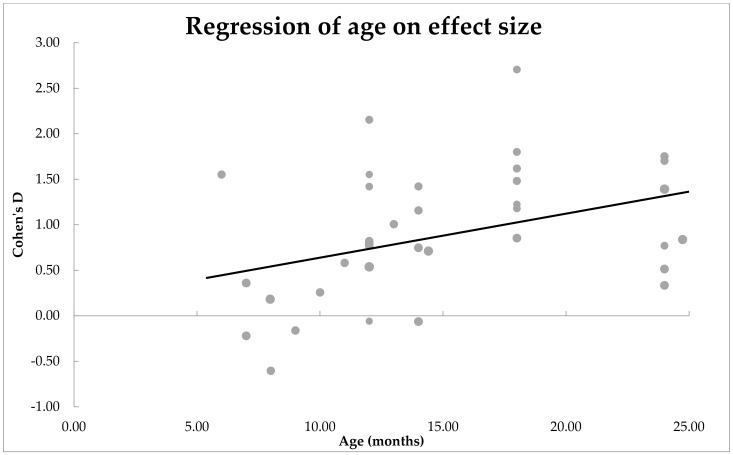
Scatterplot of how age moderated the mean difference between ASD and non-ASD infants’ expressive vocabulary. A significant effect of moderator was observed.

***Moderator analyses: Is the main effect size influenced by age of the infants?*** Age was found to be a significant moderator of effect size, *n* = 35, *QM*(1) = 7.28, *p* = 0.007. These results are shown in Figure 4 as a scatter plot. Estimates for Cohen’s *d* were mostly positive for all age groups and statistically significant from zero, *β* = 0.39, *SE* = 0.02. The positive correlation suggests that the mean difference between the two groups of infants increased with age.

***Sub-group analyses: Is the main effect size dependent on the assessment (CDI or MSEL) used?*** The effect sizes of the sub-groups of language measure (CDI and MSEL) did not significantly differ, *n* = 35, *QM*(1) = 2.68, *p* = 0.10. The CDI group was heterogenous (I^2^ = 78.60%), Cohen’s *d* = 0.68 [0.32, 1.04]. Similarly, the MSEL group was heterogenous (*I*^2^ = 75.05%), (Cohen’s *d* = 1.05 [0.76, 1.34]).

#### 3.2.2. Receptive Vocabulary

The weighted mean effect over the whole dataset was significantly above zero, with Cohen’s *d* = 0.84 [0.60, 1.09], *SE* = 0.12. The weighted mean effect, which is positive and significantly above zero, indicates that infants later diagnosed with ASD scored lower on receptive vocabulary than their TD peers.

***Publication bias.*** Following the removal of all effect sizes above 3, there was no salient evidence of publication bias. The Egger test was non-significant (*p* = 0.069) for publication bias.

***Heterogeneity.*** Heterogeneity was significant, *Q*(32) = 199.72, *p* < 0.001; total heterogeneity *I*^2^ = 83.98%. Considering this, we examined the influence of age and assessment used on the receptive vocabularies of infants diagnosed with ASD versus TD infants.

**Figure 5:**
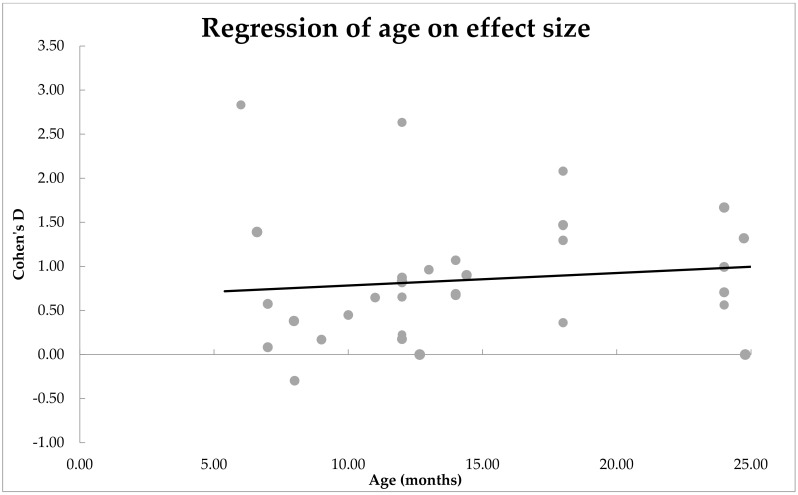
Scatterplot of how age moderated the mean difference between ASD and non-ASD infants’ receptive vocabulary. No significant effect of moderator was observed.

***Moderator analyses: Is the main effect size influenced by the age of the infants?*** Age was not found to be a significant moderator of effect size, *n* = 32, *QM*(1) = 0.45, *p* = 0.50. These results are shown in Figure 5 as a scatter plot. Estimates for Cohen’s *d* were mostly positive for all age groups, but it was not statistically significant from zero, *β* = 0.12, *SE* = 0.02. This lack of correlation suggests that the mean difference between infants with or without ASD did not increase with age.

***Sub-group analyses: Is the main effect size dependent on the assessment (CDI or MSEL) used?*** The effect sizes of the sub-groups of language measure (CDI and MSEL) did not significantly differ, *n* = 32, *QM*(1) = 2.87, *p* = 0.09. The CDI group was heterogenous (*I*^2^ = 59.74%), making it difficult to assess true main effect size, Cohen’s *d* = 0.62 [0.35, 0.90]. Similarly, the MSEL group was heterogenous (*I*^2^ = 88.66%), with no true main effect size, Cohen’s *d* = 0.99 [0.65, 1.33].

#### 3.2.3. Summary of Results: Post-Diagnostic Groups

Overall, there were large effect size observed for the studies. The direction of this effect size was that the ASD infants had smaller receptive and expressive vocabularies than their non-ASD peers. Additionally, there was substantial heterogeneity observed in effect sizes for both expressive and receptive vocabulary.

Age was found to be a moderator for expressive vocabulary size—as age increased, the mean difference between the ASD and non-ASD infants increased, with the gap in the expressive vocabulary size of the ASD and non-ASD infant groups becoming increasingly larger. In contrast, for receptive vocabulary, age was not found to be a moderator of the effect size observed.

For neither expressive nor receptive vocabulary was there a significant difference in effect sizes dependent on the language measure (CDI or MSEL) used. The assessments therefore found a similar size of difference between the ASD and non-ASD infants across studies.

## 4. Discussion

This paper aimed to examine to what extent differences existed in the expressive and receptive vocabulary sizes of infants with ASD pre-diagnostically and post-diagnostically. Pre-diagnostically, infants at elevated likelihood (EL) for ASD were compared to infants at typical likelihood (TL) for ASD. Post-diagnostically, ASD infants were compared to non-ASD infants. A systematic review and meta-analyses were carried out, aiming to compile the existing empirical research on this topic.

For both the pre-diagnostic and post-diagnostic groups, it was observed that the elevated likelihood and ASD infants had smaller expressive and receptive vocabularies than their typical likelihood and non-ASD peers. This effect size was moderate pre-diagnostically and large post-diagnostically. There was also a substantial heterogeneity both when comparing pre-diagnostic and post-diagnostic groups. Subsequently, age and language measure were assessed as moderators of the magnitude of the difference between the infant groups. In the pre-diagnostic infant groups, age was found to be a moderator of the effect size when comparing the receptive scores of the EL and TL infants. In the post-diagnostic groups, age was found to be a moderator of the effect size when comparing the expressive scores of ASD and non-ASD infants. In both instances, as the age of the infants increased, the difference between the atypical and typical groups increased. Language measure did not have an effect on the standardized mean difference between the infant groups, meaning that regardless of whether the CDI or MSEL was used, the size of the group differences was not different pre- and post-diagnostically. These results are subsequently discussed in more depth, with suggestions being made for future research directions.

### 4.1. Heterogeneity—Large Variability in the Effect Sizes across Studies

When comparing both pre- and post-diagnostic groups, there was a substantial heterogeneity observed in the effect sizes across studies. These findings were similar to those observed in other meta-analyses conducted previously on ASD populations in similar age groups [3]. A large proportion of this variability came from ‘true’ effects as opposed to random variability between participants, suggesting that there are a number of factors that could influence the vocabulary sizes of the infants. For example, language background, i.e., the proportions of monolinguals versus multilinguals tested in a study, could be one such factor.

Due to the large heterogeneity, it was not possible to interpret the weighted mean effect size of all the studies. Instead, the lower and upper 95% confidence intervals of the weighted mean effect size were interpreted. The lower confidence intervals of the weighted mean effect were positive in both pre- and post-diagnostic analyses, that is, above zero. This indicated that the atypically developing group (EL or ASD) had lower vocabulary scores than their typically developing peers (TL or non-ASD). For the pre-diagnostic groups, this was a moderate effect (expressive: *d* = 0.27; receptive; *d* = 0.29), and for the post-diagnostic results, this was a large effect (expressive: *d* = 0.65; receptive; *d* = 0.60). 

Infant group differences in vocabulary therefore appeared to be larger in post-diagnostic compared to pre-diagnostic groups. One explanation is that the group with elevated risk of ASD (pre-diagnosis) is heterogeneous; only some infants receive a diagnosis of ASD, while others do not, and the developmental profiles of the elevated likelihood infants with no diagnosis may be more similar to that of the typical likelihood infants [6,39]. In a study included in this meta-analysis, it was found that infants that are retrospectively diagnosed as ‘elevated likelihood no diagnosis’ do not differ substantially from infants that are classified prospectively as ‘typical likelihood’ [6]. The majority of ‘elevated likelihood’ infants go on to receive a diagnosis of ‘elevated likelihood no diagnosis’, meaning that there is likely to be a larger overlap in the language profile of elevated and typical likelihood infants, compared to ASD and non-ASD infants. This may be why we observed a smaller (as opposed to larger) group difference between EL and TL infant groups.

### 4.2. Does Age Moderate the Effect Size, or the Mean Difference, of the Expressive and Receptive Vocabulary Size of the Infant Groups?

**Pre-diagnostically.** The moderator analysis revealed that, for expressive vocabulary, age was not a significant moderator of effect size. However, for receptive vocabulary, age was a significant moderator of effect size. As the age of the infants increased, the effect size of difference between the EL and TL infants’ receptive vocabulary size increased. We checked whether age was equally distributed in studies that looked at expressive versus receptive vocabulary. An independent *t*-test showed that the age groups assessed were comparable. Our finding is in line with studies that have implemented other questionnaires to index language—as the infants get older, the two groups increasingly diverge from each other with regards to receptive vocabulary [40]. 

There are several possibilities as to why we observe that age moderates receptive vocabulary, but not expressive vocabularies. One interpretation for why age moderated receptive but not expressive vocabulary size is that the reliability of receptive assessments is lower for receptive vocabulary than expressive vocabulary. There could be a number of reasons for this. The parental ratings of vocabulary could be affected by their knowledge of their child’s elevated likelihood status. Since the majority of the elevated likelihood infants receive a typically developing diagnosis at 24 or 36 months, differences that are observed between the EL and TL groups may be attributable to how parents rate the child. Biases in assessment are more likely to affect receptive vocabulary ratings, which tend to have a lower reliability over time [23,41,42]. This may be why age moderates differences between the groups in receptive vocabulary size but not expressive vocabulary size.

Additionally, differences in the linguistic environment of the EL versus TL infants may also lead to differences in their vocabulary outcomes. Infants are classified as elevated likelihood because they have an older sibling with a diagnosis of ASD. We may expect familial dynamics to be different in these families where one child has a developmental disorder compared to the families of the typical likelihood infant who have an older child with no diagnosis. For example, previous research has shown that parental stress is higher in families where a child has a diagnosis of ASD [43]. This increased parental stress has been shown to affect how parents assess some aspect of their child development in child studies [44]. Although the previously mentioned study did not find stress to affect ratings of expressive and receptive vocabulary, it should be noted that this study assessed parents of older children. It could also be that infants who grow up with a sibling with ASD model their behaviour to that of their sibling and show less-ostensive reactions of word understanding. This may be why age moderates differences between the groups in receptive vocabulary size but not expressive vocabulary size.

**Post-diagnostically.** The moderator analysis revealed that age was not a significant moderator of effect size for receptive vocabulary. However, for expressive vocabulary, age was a significant moderator of effect size. As the age of the infants increased, the standardised mean difference between the ASD and non-ASD infants’ receptive vocabulary size increased. An independent *t*-test revealed that the age groups in which expressive compared to receptive vocabulary were assessed was comparable. 

An interpretation of this could be that only expressive and not receptive vocabulary is impacted in EL-ASD infants and that this was not clear when looking at the pre-diagnosis groups due to another developmental group in the EL group (such as EL-no diagnosis). Expressive vocabulary development may be more affected by the motor-related impairments that are observable in ASD children [45,46]. The motor-related difficulties that are present in children diagnosed with ASD start to affect their word production abilities prior to the age of 24 months [47]. In a study by Leonard et al. [47], the motor delays of infants that later received a diagnosis of ASD were found to predict their expressive but not receptive scores. Infants with a diagnosis of ASD may therefore only differ from their non-ASD peers in their expressive but not receptive vocabulary scores.

### 4.3. Does Language Measure Moderate the Effect Size, or the Mean Difference, of the Expressive and Receptive Vocabulary Size of the Infant Groups?

**Pre-diagnostically and post-diagnostically.** No significant differences were found in the effect size depending on whether the CDI or the MSEL was used. This indicates that the magnitude of the difference between the two groups was not influenced by how their expressive or receptive vocabulary was assessed. Both in prospective and retrospective populations, the EL and ASD groups had smaller vocabularies than their TL and non-ASD peers, respectively. This is in line with existing research that has compared the CDI and MSEL scores of children [22]. In addition, studies that have implemented other measures, such as the Reynell Developmental Language Skills, have found similar effects in the 0–24 months age groups—the language scores of ASD infants are significantly lower than that of their typical likelihood or elevated likelihood no diagnosis peers [48].

Therefore, factors such as the parents’ more extensive experience with the child do not appear to impact how accurately expressive vocabulary is assessed. Additionally, the time frame and the environment in which the assessment is done does not appear to have an impact on the effect sizes. Interestingly, when studies with an effect size of three or above were included in the meta-analysis, language measure was found to be a significant moderator of effect size. Pre-diagnostically, for receptive language, it was observed that the mean differences between the EL and TL groups were larger when they were assessed with the CDI compared to the MSEL. A statistical reason for this could be that the CDI is much longer than the MSEL and variance in the CDI as a result be larger. Another reason for the mean differences between groups being higher in the CDI could be the larger variability in how parents assess their children’s vocabulary. Whereas clinicians are required to test infants’ vocabularies in a standard format, parents rely only on their previous experience with their child. This could have led to larger variations in the scores that parents assign their infants within the CDI when compared with the scores assigned by clinicians on the MSEL. Nevertheless, we need to treat these results with caution, as findings emerged only when we included studies with very large effect sizes.

### 4.4. Limitations

The meta-analysis has some limitations. First, in our meta-analyses, we treated all collected effect sizes as *independent* effects, whereas it is unlikely that this is true. There were many studies which yielded multiple effect sizes, collected at various ages or via different methods (i.e., both CDI and Mullen). For instance, Landa and colleagues [49] followed infants with or without elevated risk of ASD and measured their vocabulary sizes at multiple time points (6, 12, and 24 months). Indeed, the majority of studies assessed vocabulary at multiple ages: For the 57 prospective studies on vocabulary development, 54% (31/57) measured vocabulary more than once. Other studies sampled vocabulary within the same children both via the CDI and via Mullen. There were nine prospective studies that reported both outcomes, such as Tran et al., [50]. In both cases, effect sizes were obviously related as they correspond to the same set of children. A third reason as to why some effect sizes are possibly related to each other is that across studies, some (parts of) datasets might have been used multiple times. Finding and testing infants at elevated likelihood of ASD is difficult, time-consuming, and costly, which is why researchers from different research sites often team up to collect data sets large enough to draw valid conclusions [4,27]. The difficulty to find subjects is also the reason why some studies first publish data prospectively, and when outcomes are known on children’s final diagnosis, also retrospectively, zooming in on those infants who are either typically developing or diagnosed with ASD. As a result, while our meta-analyses assume that all effect sizes are independent from each other, the reality is that many effect sizes are related to each other, which raises questions about the generalizability of our results and obscures true effects. Nevertheless, results indicate significant effects when we conservatively focus on the lower bound of the confidence intervals. Thus, our results suggest that across studies, there is reason to believe that infants at elevated risk of ASD develop smaller vocabularies compared to their peers.

Another limitation in our studies is while we observed heterogeneous effect sizes, there was not a perfect balance across ages or methods sampled. To illustrate, there was an imbalance in the number of papers that looked at each assessment type. In total, 75% of studies used the MSEL, whereas 16% used the CDI. There was also the case that 9% of papers tested infants on both assessment types. This, however, means that the majority of the papers were MSEL and not CDI. Additionally, there were few (if any) studies prior to 5 months of age because these time points precede what is considered the earliest stages of infants’ vocabulary comprehension [5,20].

Another limitation was that some potentially confounding factors were not controlled for when searching for and excluding papers. This includes SES and the language background of the infants. A number of studies did not have information on the SES of their participants. However, SES has frequently been shown to influence the receptive and expressive vocabulary scores of infants [51]. Language background could have also influenced the receptive and expressive vocabulary scores observed in this study. For example, a child raised in a multilingual compared to monolingual home may have had lower receptive or expressive skills in the tested language not due to developmental classification (e.g., EL or TL) but due to the frequency of exposure they had in that language. Some studies have shown that children raised in multilingual homes at certain developmental timepoints may lag behind their peers [52]. Thus, we could not examine other possible moderators that could explain the heterogeneity in effect sizes. More research on this is needed.

### 4.5. Future Research Directions

The large heterogeneity in effect sizes suggests that there are other factors contributing to the variance in the study results. Future research could therefore assess the factors leading to the heterogeneity of the effect sizes, including the SES and the language background of the infants.

Furthermore, although language measure was not found to moderate effect size, this could be attributable to studies more frequently testing older infants. Older infants are more often tested on their ability to understand and produce words and younger infants are more often tested on their ability to understand words. It therefore remains unclear as to whether language measure may moderate group differences between elevated likelihood/ASD and typical likelihood/non-ASD infants when looking at younger infants, whose vocabularies are made up of a larger proportion of words that are understood but not yet produced. Future studies could test empirically whether language measure moderates the vocabulary scores in these younger infant groups.

## 5. Conclusions

Pre-diagnostically, the lower confidence limits indicated a moderate to large effect sizes. Post-diagnostically, the lower confidence limits indicated large effect sizes. This means that larger differences were observed between the post-diagnostic (ASD vs. non-ASD) and the pre-diagnostic (EL vs. TL) group classifications. In this meta-analysis, it was found that age was a moderator on the effect size, but this effect was different pre- and post-diagnostically; whereas pre-diagnostically, age moderated receptive vocabulary only, post-diagnostically, age moderated expressive vocabulary only. These findings indicate that the developmental profiles of infants with an elevated likelihood or diagnosis of ASD diverge from that of typical likelihood or non-ASD peers. In contrast to age, language measure did not moderate effect sizes—differences between the infant groups were of similar magnitude on the CDI and MSEL.

## Figures and Tables

**Table 1:** Search in PubMed, PsychInfo, and Google Scholar.

**PubMed**	(risk[Title/Abstract] OR sibling *[Title/Abstract] OR likelihood[Title/Abstract] OR "broader autism phenotype"[Title/Abstract]) AND (ASD[Title/Abstract] OR autis *[Title/Abstract] OR asperger *[Title/Abstract] OR "pervasive develop*"[Title/Abstract]) AND ("Communicative Development Inventory"[Title/Abstract] OR CDI[Title/Abstract] OR "Mullen Scales of Early Learning"[Title/Abstract] OR MSEL[Title/Abstract] OR language[Title/Abstract] OR word*[Title/Abstract] OR vocabulary[Title/Abstract] OR communication[Title/Abstract]) AND month *[Title/Abstract]
**PsycINFO**	((risk OR sibling * OR likelihood OR "broader autism phenotype") AND (ASD OR autis * OR asperger* OR "pervasive develop *") AND ("Communicative Development Inventory" OR CDI OR "Mullen Scales of Early Learning" OR MSEL OR language OR word * OR vocabulary OR communication) AND month *).ti,ab,id
**Google Scholar**	(risk OR likelihood) AND (sibling OR siblings) AND (ASD OR autism OR asperger OR “pervasive developmental”) AND (“Communicative Development Inventory” OR “Mullen Scales of Early Learning”) AND (month OR months)

**Table 2:** Inclusion and exclusion criteria for the search.

Inclusion Criteria	Exclusion Criteria
RCT, cohort, cross-sectional or case-control study	All other study designs (e.g., reviews)
Language scores of CDI or MSEL as outcome measures	Outcome different from CDI or MSEL
Compares language scores of EL or ASD group with TL or non-ASD groups, respectively	Does not compare language scores of ASD or EL infants with TL or non-ASD infants
Language scores measured between 0 and 24 months	Language measure only out of the range of 0 to 24 months
Full text available	
Text in English	

Note: ASD = autism spectrum disorder; EL = elevated likelihood for autism; TD = typical development; TL = typical likelihood for autism; RCT = random control trial; MSEL = Mullen Scales of Early Learning [20]; CDI = MacArthur–Bates Communicative Development Inventory [5].

## Data Availability

Not applicable.

## Appendix A

**Table A1:** Results from studies without clinical diagnosis on the Communicative Development Inventory.

Reference	Ages of Testing (Months)	Average Mumber of Participants *	Both Expressive and Receptive?
Bontinck et al., 2018 [53]	24	32 EL, 24 TL	Both
Curtin and Vouloumanos, 2013 [54]	12, 18	31 EL, 31 TL	Both
Drouker et al., 2013 [55]	12, 18	13 EL, 21 TL	Only expressive
Edmunds et al., 2017 [56]	12, 15, 18	50 EL, 34 TL	Only expressive
Edwards, 2016 [57]	18	7 EL, 6 TL	Both
Ference and Curtin, 2013 [58]	12	19 EL, 19 TL	Both
Ference and Curtin, 2015 [59]	12	20 EL, 23 TL	Both
Sperle, 2019 [60]	24	47 EL, 39 TL	Only expressive
Stone et al., 2007 [61]	12–23	64 EL, 42 TL	Both
Tran, 2020 [50]	18	36 EL, 27 TL	Both

* Averaged across age groups.

**Table A2:** Studies without clinical diagnosis on the Mullen Scales of Early Learning.

Reference	Ages of Testing (Months)	Average Number of Participants *	Both Expressive and Receptive Measured?
Bontinck et al., 2018 [53]	24	32 EL, 24 TL	Both
Bruyneel et al., 2019a [62]	10, 14	32 EL, 31 TL	Both
Drouker et al., 2013 [55]	12	14 EL, 22 TL	Only expressive
Edwards, 2016 [57]	18	21 EL, 17 TL	Both
Franchini et al., 2018 [63]	6	482 EL, 178 TL	Both
Gangi et al., 2014 [64]	24	41 EL, 17 TL	Both
Kadlaskar et al., 2020 [65]	12	31 EL, 27 TL	Both
Mulligan et al., 2012 [8]	12	13 EL, 12 TL	Both
Nyström et al., 2015 [66]	10	29 EL, 15 TL	Both
Paul et al., 2011 [11]	6, 12, 24	24 EL, 21 TL	Both
Righi et al., 2014 [19]	6, 12	28 EL, 26 TL	Both
Seery et al., 2013 [67]	6, 12	62 EL, 46 TL	Both
Seery et al., 2014 [68]	6, 12, 18	35 EL, 45 TL	Both
Srinivasan et al., 2020 [69]	11, 15	16 EL, 16 TL	Both
Stone et al., 2007 [61]	12–23	64 EL, 42 TL	Both
Swanson et al., 2018 [70]	6–9	40 EL, 19 TL	Both
Tran, 2020 [50]	18	36 EL, 27 TL	Both
Unwin et al., 2017 [71]	12, 24	22 EL, 27 TL	Both
Young et al., 2009 [72]	24	33 EL, 25 TL	Both

* Averaged across age groups.

**Table A3:** Studies with clinical diagnosis on the MacArthur–Bates Communicative Development Inventory.

Reference	Ages of Testing (Months)	Average Number of Participants *	Both Expressive and Receptive Measured?
Hudry et al., 2014 [67]	7, 14, 24	17 ASD, 48 non-ASD	Both
Iverson et al., 2018 [6]	8, 9, 10, 11, 12, 13, 14, 18, 24	41 ASD, 28 non-ASD	Both
Lazenby et al., 2016 [73]	12	43 ASD, 133 non-ASD	Both
Mitchell et al., 2006 [74]	12, 18	15 ASD, 49 non-ASD	Both
Roemer et al., 2019 [75]	12	14 ASD, 25 non-ASD	Only expressive
Roemer et al., 2019 [75]	14, 18	14 ASD, 25 non-ASD	Both
Zwaigenbaum et al., 2005 [76]	12	4 ASD, 12 non-ASD	Both

* Averaged across age groups.

**Table A4:** Studies with clinical diagnosis on the Mullen Scales of Early Learning.

Reference	Ages of Testing (Months)	Average Number of Participants *	Both Expressive and Receptive Measured?
Hudry et al., 2014 [17]	7, 14, 24	17 ASD, 48 non-ASD	Both
Bacon and Catherine, 2015 [77]	12, 14, 18, 24	69 ASD, 130 non-ASD	Both
Bussu et al., 2018 [78]	8, 14, 24	32 ASD, 71 non-ASD	Both
Chawarska et al., 2013 [79]	6, 24	12 ASD, 35 non-ASD	Both
Chawarska et al., 2014 [80]	18	157 ASD, 348 non-ASD	Only expressive
Chenausky et al., 2017 [81]	12, 18, 24	10 ASD, 18 non-ASD	Both
Estes et al., 2015 [82]	6, 12, 18	49 ASD, 98 non-ASD	Both
Landa et al., 2007 [49]	6, 14, 24	23 ASD, 53 non-ASD	Both
Lazenby et al., 2016 [73]	12	43 ASD, 133 non-ASD	Both
Levin et al., 2017 [83]	12, 18, 24	7 ASD, 14 non-ASD	Both
Libertus et al., 2014 [84]	6	22 ASD, 22 non-ASD	Both
Macari et al., 2012 [85]	12, 18, 24	13 ASD, 34 non-ASD	Both
Meera et al., 2020 [86]	12, 24	54 ASD, 42 non-ASD	Both
Messinger et al., 2015 [87]	6, 12, 18, 24	252 ASD, 583 non-ASD	Both
Ozonoff et al., 2010 [4]	6, 12, 18, 24	25 ASD, 25 non-ASD	Both
Ozonoff et al., 2014 [39]	6, 12, 18, 24	51 ASD, 116 non-ASD	Both
Pijl et al., 2019 [88]	8, 14	24 ASD, 66 non-ASD	Both
Seery, 2015 [89]	6, 12, 18, 24	13 ASD, 36 non-ASD	Both
Swanson et al., 2017 [90]	6, 12, 24	13 ASD, 29 non-ASD	Both
Talbott, 2015 [18]	12	6 ASD, 30 non-ASD	Only expressive
Wagner et al., 2018 [91]	18	6 ASD, 32 non-ASD	Both
Zwaigenbaum et al., 2005 [76]	12	4 ASD, 12 non-ASD	Both

* Averaged across age groups.

## References

[1] American Psychiatric Association (2013). Diagnostic and Statistical Manual of Mental Disorders: DSM-5.

[2] Jones E.J.H., Gliga T., Bedford R., Charman T., Johnson M.H. (2014). Developmental Pathways to Autism: A Review of Prospective Studies of Infants at Risk. Neurosci. Biobehav. Rev..

[3] Kwok E.Y.L., Brown H.M., Smyth R.E., Oram Cardy J. (2015). Meta-Analysis of Receptive and Expressive Language Skills in Autism Spectrum Disorder. Res. Autism Spectr. Disord..

[4] Ozonoff S., Young G.S., Carter A., Messinger D., Yirmiya N., Zwaigenbaum L., Bryson S., Carver L.J., Constantino J.N., Dobkins K. (2011). Recurrence Risk for Autism Spectrum Disorders: A Baby Siblings Research Consortium Study. Pediatrics.

[5] Fenson L., Dale P.S., Reznick J.S., Bates E., Thal D.J., Pethick S.J., Tomasello M., Mervis C.B., Stiles J. (1994). Variability in Early Communicative Development. Monogr. Soc. Res. Child Dev..

[6] Iverson J.M., Northrup J.B., Leezenbaum N.B., Parladé M.V., Koterba E.A., West K.L. (2018). Early Gesture and Vocabulary Development in Infant Siblings of Children with Autism Spectrum Disorder. J. Autism Dev. Disord..

[7] Miller M., Iosif A.-M., Hill M., Young G.S., Schwichtenberg A.J., Ozonoff S. (2017). Response to Name in Infants Developing Autism Spectrum Disorder: A Prospective Study. J. Pediatrics.

[8] Mulligan S., White B.P. (2012). Sensory and Motor Behaviors of Infant Siblings of Children with and without Autism. Am. J. Occup. Ther..

[9] Nyström P., Thorup E., Bölte S., Falck-Ytter T. (2019). Joint Attention in Infancy and the Emergence of Autism. Biol. Psychiatry.

[10] Osterling J., Dawson G. (1994). Early Recognition of Children with Autism: A Study of First Birthday Home Videotapes. J. Autism Dev. Disord..

[11] Paul R., Fuerst Y., Ramsay G., Chawarska K., Klin A. (2011). Out of the Mouths of Babes: Vocal Production in Infant Siblings of Children with ASD. J. Child Psychol. Psychiatry Allied Discip..

[12] Pickles A., Simonoff E., Conti-Ramsden G., Falcaro M., Simkin Z., Charman T., Chandler S., Loucas T., Baird G. (2009). Loss of Language in Early Development of Autism and Specific Language Impairment. J. Child Psychol. Psychiatry.

[13] Tenenbaum E.J., Amso D., Abar B., Sheinkopf S.J. (2014). Attention and Word Learning in Autistic, Language Delayed and Typically Developing Children. Front. Psychol..

[14] Veness C., Prior M., Bavin E., Eadie P., Cini E., Reilly S. (2012). Early Indicators of Autism Spectrum Disorders at 12 and 24 Months of Age: A Prospective, Longitudinal Comparative Study. Autism.

[15] Werner E., Dawson G., Munson J., Osterling J. (2005). Variation in Early Developmental Course in Autism and Its Relation with Behavioral Outcome at 3–4 Years of Age. J. Autism Dev. Disord..

[16] Nyström P., Bölte S., Falck-Ytter T., EASE Team (2017). Responding to Other People’s Direct Gaze: Alterations in Gaze Behavior in Infants at Risk for Autism Occur on Very Short Timescales. J. Autism Dev. Disord..

[17] Hudry K., Chandler S., Bedford R., Pasco G., Gliga T., Elsabbagh M., Johnson M.H., Charman T. (2014). Early Language Profiles in Infants at High-Risk for Autism Spectrum Disorders. J. Autism Dev. Disord..

[18] Talbott M.R., Nelson C.A., Tager-Flusberg H. (2015). Maternal Gesture Use and Language Development in Infant Siblings of Children with Autism Spectrum Disorder. J. Autism Dev. Disord..

[19] Righi G., Tierney A.L., Tager-Flusberg H., Nelson C.A. (2014). Functional Connectivity in the First Year of Life in Infants at Risk for Autism Spectrum Disorder: An EEG Study. PLoS ONE.

[20] Bradley-Johnson S. (1997). Mullen Scales of Early Learning. Psychol. Sch..

[21] Lee E.B., Volkmar F.R. (2013). Mullen Scales of Early Learning. Encyclopedia of Autism Spectrum Disorders.

[22] Nordahl-Hansen A., Kaale A., Ulvund S.E. (2013). Inter-Rater Reliability of Parent and Preschool Teacher Ratings of Language in Children with Autism. Res. Autism Spectr. Disord..

[23] Tomasello M., Mervis C.B. (1994). The Instrument Is Great, but Measuring Comprehension Is Still a Problem. Monogr. Soc. Res. Child Dev..

[24] Houston-Price C., Mather E., Sakkalou E. (2007). Discrepancy between Parental Reports of Infants’ Receptive Vocabulary and Infants’ Behaviour in a Preferential Looking Task. J. Child Lang..

[25] Styles S., Plunkett K. (2009). What Is ‘Word Understanding’ for the Parent of a One-Year-Old? Matching the Difficulty of a Lexical Comprehension Task to Parental CDI Report. J. Child Lang..

[26] Balasundaram P., Avulakunta I.D. (2022). Bayley Scales Of Infant and Toddler Development. StatPearls.

[27] Jones E.J.H., Mason L., Begum Ali J., van den Boomen C., Braukmann R., Cauvet E., Demurie E., Hessels R.S., Ward E.K., Hunnius S. (2019). Eurosibs: Towards Robust Measurement of Infant Neurocognitive Predictors of Autism across Europe. Infant Behav. Dev..

[28] Hazlett H.C., Gu H., McKinstry R.C., Shaw D.W., Botteron K.N., Dager S.R., Styner M., Vachet C., Gerig G., Paterson S.J. (2012). Brain Volume Findings in 6-Month-Old Infants at High Familial Risk for Autism. Am. J. Psychiatry.

[29] Bishop S.L., Guthrie W., Coffing M., Lord C. (2011). Convergent Validity of the Mullen Scales of Early Learning and the Differential Ability Scales in Children with Autism Spectrum Disorders. Am. J. Intellect. Dev. Disabil..

[30] Mayor J., Plunkett K. (2011). A Statistical Estimate of Infant and Toddler Vocabulary Size from CDI Analysis. Dev. Sci..

[31] Anderson D.K., Lord C., Risi S., DiLavore P.S., Shulman C., Thurm A., Welch K., Pickles A. (2007). Patterns of Growth in Verbal Abilities among Children with Autism Spectrum Disorder—PsycNET. J. Consult. Clin. Psychol..

[32] Ouzzani M., Hammady H., Fedorowicz Z., Elmagarmid A. (2016). Rayyan—A Web and Mobile App for Systematic Reviews. Syst. Rev..

[33] Moher D., Liberati A., Tetzlaff J., Altman D.G., PRISMA Group (2009). Preferred Reporting Items for Systematic Reviews and Meta-Analyses: The PRISMA Statement. PLoS Med..

[34] MetaLab. https://langcog.github.io/metalab/.

[35] Tsuji S., Bergmann C., Lewis M., Braginsky M., Piccinini P., Frank M.C., Cristia A. (2017). MetaLab: A Repository for Meta-Analyses on Language Development, and More. Interspeech.

[36] Suurmond R., van Rhee H., Hak T. (2017). Introduction, Comparison, and Validation of Meta-Essentials: A Free and Simple Tool for Meta-analysis—Suurmond—2017—Research Synthesis Methods—Wiley Online Library. Res. Synth. Methods.

[37] Hak T., van Rhee H., Suurmond R. (2016). How to Interpret Results of Meta-Analysis.

[38] Van Rhee H., Suurmond R., Hak T. (2015). User Manual for Meta-Essentials: Workbooks for Meta-Analysis.

[39] Ozonoff S., Young G.S., Belding A., Hill M., Hill A., Hutman T., Johnson S., Miller M., Rogers S.J., Schwichtenberg A.J. (2014). The Broader Autism Phenotype in Infancy: When Does It Emerge?. J. Am. Acad. Child Adolesc. Psychiatry.

[40] Gamliel I., Yirmiya N., Sigman M. (2007). The Development of Young Siblings of Children with Autism from 4 to 54 Months. J. Autism Dev. Disord..

[41] Luyster R.J., Kadlec M.B., Carter A., Tager-Flusberg H. (2008). Language Assessment and Development in Toddlers with Autism Spectrum Disorders. J. Autism Dev. Disord..

[42] Sachse S., Suchodoletz W.V. (2008). Early Identification of Language Delay by Direct Language Assessment or Parent Report?. J. Dev. Behav. Pediatrics.

[43] Schieve L.A., Blumberg S.J., Rice C., Visser S.N., Boyle C. (2007). The Relationship Between Autism and Parenting Stress. Pediatrics.

[44] Schwartzman J.M., Hardan A.Y., Gengoux G.W. (2021). Parenting Stress in Autism Spectrum Disorder May Account for Discrepancies in Parent and Clinician Ratings of Child Functioning. Autism.

[45] Iverson J.M. (2010). Developing Language in a Developing Body: The Relationship between Motor Development and Language Development. J. Child Lang..

[46] Oudgenoeg-Paz O., Volman M.J.C., Leseman P.P. (2012). Attainment of Sitting and Walking Predicts Development of Productive Vocabulary between Ages 16 and 28 Months—ScienceDirect. Infant Behav. Dev..

[47] Leonard H.C., Bedford R., Pickles A., Hill E.L. (2015). Predicting the Rate of Language Development from Early Motor Skills in At-Risk Infants Who Develop Autism Spectrum Disorder. Res. Autism Spectr. Disord..

[48] Gamliel I., Yirmiya N., Jaffe D.H., Manor O., Sigman M. (2009). Developmental Trajectories in Siblings of Children with Autism: Cognition and Language from 4 Months to 7 Years. J. Autism Dev. Disord..

[49] Landa R. (2007). Early Communication Development and Intervention for Children with Autism. Ment. Retard. Dev. Disabil. Res. Rev..

[50] Tran X.A. (2020). Neural Connectivity in Infants at Familial Risk for Autism Spectrum Disorder.

[51] Hoff E. (2006). How Social Contexts Support and Shape Language Development. Dev. Rev..

[52] Kovács Á.M., Nicoladis E., Montanari S., Nicoladis E., Montanari S. (2016). Chapter 13 Cognitive Effects of Bilingualism in Infancy.

[53] Bontinck C., Warreyn P., Demurie E., Bruyneel E., Boterberg S., Roeyers H. (2018). Social Interactions Between 24-Month-Old Children and Their Older Sibling with Autism Spectrum Disorder: Characteristics and Association with Social-Communicative Development. J. Autism Dev. Disord..

[54] Curtin S., Vouloumanos A. (2013). Speech Preference Is Associated with Autistic-Like Behavior in 18-Months-Olds at Risk for Autism Spectrum Disorder. J. Autism Dev. Disord..

[55] Droucker D., Curtin S., Vouloumanos A. (2013). Linking Infant-Directed Speech and Face Preferences to Language Outcomes in Infants at Risk for Autism Spectrum Disorder. J. Speech Lang. Hear. Res. JSLHR.

[56] Edmunds S.R., Ibañez L.V., Warren Z., Messinger D.S., Stone W.L. (2017). Longitudinal Prediction of Language Emergence in Infants at High and Low Risk for Autism Spectrum Disorder. Dev. Psychopathol..

[57] Edwards L.A. (2016). Neural Precursors of Language in Infants at High Risk for Autism Spectrum Disorder. Ph.D. Thesis.

[58] Ference J., Curtin S. (2013). Attention to Lexical Stress and Early Vocabulary Growth in 5-Month-Olds at Risk for Autism Spectrum Disorder. J. Exp. Child Psychol..

[59] Ference J., Curtin S. (2015). The Ability to Map Differentially Stressed Labels to Objects Predicts Language Development at 24 Months in 12-Month-Olds at High Risk for Autism. Infancy.

[60] Sperle L. (2019). Visual Attention Composites across the Early Development of Infants at Heightened Genetic Risk for Autism Spectrum Disorder. Ph.D. Thesis.

[61] Stone W.L., McMahon C.R., Yoder P.J., Walden T.A. (2007). Early Social-Communicative and Cognitive Development of Younger Siblings of Children with Autism Spectrum Disorders. Arch. Pediatrics Adolesc. Med..

[62] Bruyneel E., Demurie E., Warreyn P., Roeyers H. (2019). The Mediating Role of Joint Attention in the Relationship between Motor Skills and Receptive and Expressive Language in Siblings at Risk for Autism Spectrum Disorder. Infant Behav. Dev..

[63] Franchini M., Duku E., Armstrong V., Brian J., Bryson S.E., Garon N., Roberts W., Roncadin C., Zwaigenbaum L., Smith I.M. (2018). Variability in Verbal and Nonverbal Communication in Infants at Risk for Autism Spectrum Disorder: Predictors and Outcomes. J. Autism Dev. Disord..

[64] Gangi D.N., Ibañez L.V., Messinger D.S. (2014). Joint Attention Initiation with and without Positive Affect: Risk Group Differences and Associations with ASD Symptoms. J. Autism Dev. Disord..

[65] Kadlaskar G., Seidl A., Tager-Flusberg H., Nelson C.A., Keehn B. (2020). Caregiver Touch-Speech Communication and Infant Responses in 12-Month-Olds at High Risk for Autism Spectrum Disorder. J. Autism Dev. Disord..

[66] Nyström P., Gredebäck G., Bölte S., Falck-Ytter T. (2015). Hypersensitive Pupillary Light Reflex in Infants at Risk for Autism. Mol. Autism.

[67] Seery A.M., Vogel-Farley V., Tager-Flusberg H., Nelson C.A. (2013). Atypical Lateralization of ERP Response to Native and Non-Native Speech in Infants at Risk for Autism Spectrum Disorder. Dev. Cogn. Neurosci..

[68] Seery A., Tager-Flusberg H., Nelson C.A. (2014). Event-Related Potentials to Repeated Speech in 9-Month-Old Infants at Risk for Autism Spectrum Disorder. J. Neurodev. Disord..

[69] Srinivasan S., Bhat A. (2020). Differences in Caregiver Behaviors of Infants At-Risk for Autism and Typically Developing Infants from 9 to 15 Months of Age. Infant Behav. Dev..

[70] Swanson M.R., Shen M.D., Wolff J.J., Boyd B., Clements M., Rehg J., Elison J.T., Paterson S., Parish-Morris J., Chappell J.C. (2018). Naturalistic Language Recordings Reveal “Hypervocal” Infants at High Familial Risk for Autism. Child Dev..

[71] Unwin L.M., Bruz I., Maybery M.T., Reynolds V., Ciccone N., Dissanayake C., Hickey M., Whitehouse A.J. (2017). Acoustic Properties of Cries in 12-Month Old Infants at High-Risk of Autism Spectrum Disorder. J. Autism Dev. Disord..

[72] Young G.S., Merin N., Rogers S.J., Ozonoff S. (2009). Gaze Behavior and Affect at 6 Months: Predicting Clinical Outcomes and Language Development in Typically Developing Infants and Infants at Risk for Autism. Dev. Sci..

[73] Lazenby D.C., Sideridis G.D., Huntington N., Prante M., Dale P.S., Curtin S., Henkel L., Iverson J.M., Carver L., Dobkins K. (2016). Language Differences at 12 Months in Infants Who Develop Autism Spectrum Disorder. J. Autism Dev. Disord..

[74] Mitchell S., Brian J., Zwaigenbaum L., Roberts W., Szatmari P., Smith I., Bryson S. (2006). Early Language and Communication Development of Infants Later Diagnosed with Autism Spectrum Disorder. J. Dev. Behav. Pediatrics.

[75] Roemer E.J., West K.L., Northrup J.B., Iverson J.M. (2019). Word Comprehension Mediates the Link between Gesture and Word Production: Examining Language Development in Infant Siblings of Children with Autism Spectrum Disorder. Dev. Sci..

[76] Zwaigenbaum L., Bryson S., Rogers T., Roberts W., Brian J., Szatmari P. (2005). Behavioral Manifestations of Autism in the First Year of Life. Int. J. Dev. Neurosci..

[77] Bacon E.C. (2014). Investigation of Early Symptom Presentation in Children under Age Three with Risk for Autism. Ph.D. Thesis.

[78] Bussu G., Jones E.J.H., Charman T., Johnson M.H., Buitelaar J.K., Team B. (2018). Prediction of Autism at 3 Years from Behavioural and Developmental Measures in High-Risk Infants: A Longitudinal Cross-Domain Classifier Analysis. J. Autism Dev. Disord..

[79] Chawarska K., Macari S., Shic F. (2013). Decreased Spontaneous Attention to Social Scenes in 6-Month-Old Infants Later Diagnosed with Autism Spectrum Disorders. Biol. Psychiatry.

[80] Chawarska K., Shic F., Macari S., Campbell D.J., Brian J., Landa R., Hutman T., Nelson C.A., Ozonoff S., Tager-Flusberg H. (2014). 18-Month Predictors of Later Outcomes in Younger Siblings of Children with Autism Spectrum Disorder: A Baby Siblings Research Consortium Study. J. Am. Acad. Child Adolesc. Psychiatry.

[81] Chenausky K., Nelson C., Tager-Flusberg H. (2017). Vocalization Rate and Consonant Production in Toddlers at High and Low Risk for Autism. J. Speech Lang. Hear. Res. JSLHR.

[82] Estes A., Zwaigenbaum L., Gu H., St John T., Paterson S., Elison J.T., Hazlett H., Botteron K., Dager S.R., Schultz R.T. (2015). Behavioral, Cognitive, and Adaptive Development in Infants with Autism Spectrum Disorder in the First 2 Years of Life. J. Neurodev. Disord..

[83] Levin A.R., Varcin K.J., O’Leary H.M., Tager-Flusberg H., Nelson C.A. (2017). EEG Power at 3 Months in Infants at High Familial Risk for Autism. J. Neurodev. Disord..

[84] Libertus K., Sheperd K.A., Ross S.W., Landa R.J. (2014). Limited Fine Motor and Grasping Skills in 6-Month-Old Infants at High Risk for Autism. Child Dev..

[85] Macari S.L., Campbell D., Gengoux G.W., Saulnier C.A., Klin A.J., Chawarska K. (2012). Predicting Developmental Status from 12 to 24 Months in Infants at Risk for Autism Spectrum Disorder: A Preliminary Report. J. Autism Dev. Disord..

[86] Meera S.S., Donovan K., Wolff J.J., Zwaigenbaum L., Elison J.T., Kinh T., Shen M.D., Estes A.M., Hazlett H.C., Watson L.R. (2020). Towards a Data Driven Approach to Screen for Autism Risk at 12 Months of Age. J. Am. Acad. Child Adolesc. Psychiatry.

[87] Messinger D.S., Young G.S., Webb S.J., Ozonoff S., Bryson S.E., Carter A., Carver L., Charman T., Chawarska K., Curtin S. (2015). Early Sex Differences Are Not Autism-Specific: A Baby Siblings Research Consortium (BSRC) Study. Mol. Autism.

[88] Pijl M.K.J., Bussu G., Charman T., Johnson M.H., Jones E.J., Pasco G., Oosterling I.J., Rommelse N.N.J., Buitelaar J.K., Team B. (2019). Temperament as an Early Risk Marker for Autism Spectrum Disorders? A Longitudinal Study of High-Risk and Low-Risk Infants. J. Autism Dev. Disord..

[89] Seery A.M. (2015). Electrophysiological Indices of Language Processing in Infants at Risk for Asd. Ph.D. Thesis.

[90] Swanson M.R., Shen M.D., Wolff J.J., Elison J.T., Emerson R.W., Styner M.A., Hazlett H.C., Truong K., Watson L.R., Paterson S. (2017). Subcortical Brain and Behavior Phenotypes Differentiate Infants With Autism Versus Language Delay. Biol. Psychiatry. Cogn. Neurosci. Neuroimaging.

[91] Wagner J.B., Luyster R.J., Moustapha H., Tager-Flusberg H., Nelson C.A. (2018). Differential Attention to Faces in Infant Siblings of Children with Autism Spectrum Disorder and Associations with Later Social and Language Ability. Int. J. Behav. Dev..

